# A pairwise strategy for imputing predictive features when combining multiple datasets

**DOI:** 10.1093/bioinformatics/btac839

**Published:** 2022-12-28

**Authors:** Yujie Wu, Boyu Ren, Prasad Patil

**Affiliations:** Department of Biostatistics, Harvard T.H. Chan School of Public Health, Boston, MA 02115, USA; Laboratory for Psychiatric Biostatistics, McLean Hospital, Belmont, MA 02478, USA; Department of Psychiatry, Harvard Medical School, Boston, MA 02115, USA; Department of Biostatistics, Boston University School of Public Health, Boston, MA 02118, USA

## Abstract

**Motivation:**

In the training of predictive models using high-dimensional genomic data, multiple studies’ worth of data are often combined to increase sample size and improve generalizability. A drawback of this approach is that there may be different sets of features measured in each study due to variations in expression measurement platform or technology. It is often common practice to work only with the intersection of features measured in common across all studies, which results in the blind discarding of potentially useful feature information that is measured in individual or subsets of studies.

**Results:**

We characterize the loss in predictive performance incurred by using only the intersection of feature information available across all studies when training predictors using gene expression data from microarray and sequencing datasets. We study the properties of linear and polynomial regression for imputing discarded features and demonstrate improvements in the external performance of prediction functions through simulation and in gene expression data collected on breast cancer patients. To improve this process, we propose a pairwise strategy that applies any imputation algorithm to two studies at a time and averages imputed features across pairs. We demonstrate that the pairwise strategy is preferable to first merging all datasets together and imputing any resulting missing features. Finally, we provide insights on which subsets of intersected and study-specific features should be used so that missing-feature imputation best promotes cross-study replicability.

**Availability and implementation:**

The code is available at https://github.com/YujieWuu/Pairwise_imputation.

**Supplementary information:**

Supplementary information is available at *Bioinformatics* online.

## 1 Introduction

Individual gene expression profiles have successfully been used to model the prognosis or risk of many diseases and disorders in personalized medicine (van ’t Veer *et al.*, 2002; Wang *et al.*, 2005). These predictive models capture disease identification (Fakoor *et al.*, 2013; Tan and Gilbert, 2003), cancer subtyping (Gao *et al.*, 2019; Huang *et al.*, 2018) and risks of recurrence and relapse (Ascierto *et al.*, 2012; Hartmann *et al.*, 2005; Wang *et al.*, 2004). Technological advancements have seen these studies graduate from custom chips to commercial tools to whole-genome sequencing. This has yielded larger-scale experiments and, over time, the ability to combine multiple gene expression studies of the same disease outcome measured in different patient cohorts. This abundance of data has led to the use of complex statistical prediction and machine-learning algorithms for the development of gene signatures (Pirooznia *et al.*, 2008; Shipp *et al.*, 2002; Ye *et al.*, 2003).

A critical issue facing the translation of these gene signatures into viable clinical tests is generalizability, or how well we expect the predictor to perform on a new patient or set of patients. Techniques such as cross-validation can overestimate how well a prediction model will generalize as compared with direct evaluation in a held-out test or validation dataset (Bernau *et al.*, 2014). This discrepancy is often due to cross-study heterogeneity in patient characteristics, measurement platforms and study designs (Patil and Parmigiani, 2018).

To combat the effects of cross-study heterogeneity and increase the training sample size to improve generalization, researchers have merged multiple studies (Xu *et al.*, 2008). van Vliet *et al.* (2008) showed that pooling datasets together will result in more accurate classification and convergence of signature genes. However, a major challenge in combining these datasets is that the same gene features may not be measured across all studies. This may be due to differences in measurement platform or variations in the same platform when studies are conducted at different points in time.

A common strategy when faced with differing sets of measured genes across studies is to retain only the intersection of gene features found in all studies (Xu *et al.*, 2005). We henceforth refer to this method of aggregation as ‘omitting’, because it simply omits gene expression information that is not measured in at least one study. Taminau *et al.* (2014) proposed a detailed procedure for merging datasets by taking the intersected genes of all studies followed by a batch effect removal procedure. Although omitting provides a simple approach for seamlessly merging studies, it comes with the potentially high cost of discarding important predictive information in features not contained in the intersection. Yasrebi *et al.* (2009) noted that if some genes that have high diagnostic power are not available for all studies, the aggregated data may not actually improve the final predictive model.

A solution to the data loss due to omission is imputation. Zhou *et al.* (2017) built LASSO models to impute missing genes across different studies assayed by two Affymetrix platforms for which the probe names of one platform are a proper subset of the other. Bobak *et al.* (2020) built several imputation models across studies that are measured using a variety of gene expression platforms. Both approaches proceed by first merging all studies together, then using the common genes in the intersection to impute missing genes. As the number of studies increases, the size of the intersection is likely to decrease, resulting in a smaller candidate feature pool and less accurate imputation of omitted genes. This makes merging before imputing a less attractive option when dealing with more than two studies, such as in the cases of the *MetaGxData*, *CuratedOvarianData* or *CuratedBreastData* collections where dozens of datasets may be available for combination (Ganzfried *et al.*, 2013; Gendoo *et al.*, 2019; Planey and Butte, 2013). Moreover, these previous approaches were focused on the accurate imputation of missing genes and its effect on downstream analyses such as gene pathway enrichment analysis. Whether or not imputation can help improve a prediction model and make it more generalizable to external data in this context is mostly unstudied.

In this article, we propose a pairwise strategy, in which instead of merging all available studies together at the outset to build an imputation model for missing gene features, we merge two studies at a time and perform imputation within the pair. We then repeat this imputation procedure for all possible pairs of studies and average imputed values for features that are missing across multiple pairs before training a prediction model. Inspired by the concept of *knowledge transfer* proposed by Vapnik and Izmailov (2015), which posits that some functional forms of the existing features could potentially capture missing information, we examine the ability of both linear and polynomial regression to impute missing features and use LASSO models (Hastie *et al.*, 2009) both for imputation and for outcome prediction. Our strategy can be implemented using any imputation method applicable to the data types being studied, and we evaluate both traditional and machine learning-based imputation methods. Lastly, we consider the impact of using only features selected as ‘important’ (highly associated with the predictive outcome of interest) across both studies within a given study pair (‘Core Imputation’) versus using all available features (‘All Imputation’) when building study-specific imputation models. Here, we revisit the question of whether some form of feature selection and a resulting smaller and more focused set of candidate features is preferable to applying regularization to a larger set of candidate features (Demir-Kavuk *et al.*, 2011; Spooner *et al.*, 2020).

The article is organized as follows: Section 2 presents formal notation for the general pairwise strategy to impute study-specific missing genes across multiple studies, as well as the specific ‘Core’ and ‘All’ methods. Section 3 presents a simulation study that evaluates the performance of the pairwise strategy and the ‘Core’ and ‘All’ methods. Section 4 describes a real data analysis predicting the expression of the gene ESR1 across multiple curated breast cancer studies. Section 5 concludes with a discussion.

## 2 Materials and methods

### 2.1 Problem statement

Let s=1,2,…,S index the studies for aggregated analysis, with *n_s_* individuals and *p_s_* genes in study *s*. Let $X_{s}$ denote the gene expression dataset for the *s*th study. $X_{s}$ is a $n_{s}\times p_{s}$ matrix where each row represents an individual and each column represents the measurement values for a particular gene. Let $Y_{s}$ be a $n_{s}\times 1$ column vector of the response variable of the *s*th study. For a pair of studies *s* and *j*, denote by $G_{sj},\;G_{s/j}$ and $G_{j/s}$ the set of genes that are found in both studies, unique to study *s* and unique to study *j*, respectively. Let $|G_{sj}|=p_{sj}$, where |·| is the cardinality of a set. It follows that $|G_{s/j}|=p_{s}-p_{sj}$ and $|G_{j/s}|=p_{j}-p_{sj}$. Denote the gene expression matrix for a subset of genes G in study *s* as $X_{s,G}$. Throughout the article, we assume that *p_sj_* > 0 for every pair of (*s*, *j*) (complete notation table in Supplementary material S1).

**Fig. 1.**
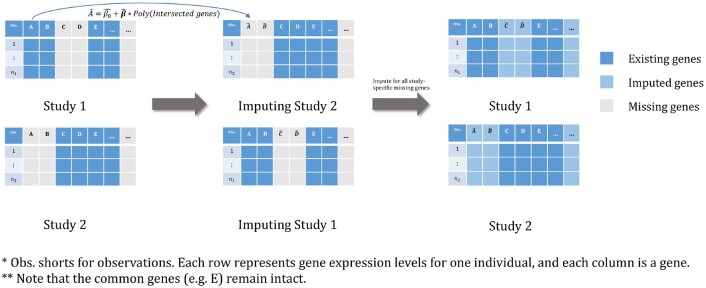
Flow chart of the pairwise imputation approach for study-specific missing genes

Our goal is to impute study-specific missing genes to augment the candidate gene set used for building a predictive model. To this end, we propose a pairwise approach where imputation is applied for two studies at a time, as the available intersection of genes across any two studies will tend to be larger than that across all *S* studies. For studies *s* and *j*, the pairwise approach uses $G_{sj}$ to construct imputation models of every gene in $G_{s/j}$ separately using data from study *s*, based on which the expression profiles in $G_{s/j}$ will be imputed for study *j*. The same procedure applies to the imputation of genes in $G_{j/s}$ for study *s*. The imputed studies *s* and *j* both then contain the same set of genes $G_{s}\cup G_{j}$ (see Fig. 1). We repeat this pairwise approach for all $\left(\begin{array}{c} S\\ 2 \end{array}\right)$ pairs of studies. If a particular gene in one study is imputed multiple times across pairs, we average its imputed values over all imputations. The result from a single imputation may be overfit to its pair and may not generalize well across pairs. We follow the philosophy of ensemble modeling (Zhang and Ma, 2012) which suggests that averaging across pairs will limit overfitting and outperform the single-best imputation model. Training only with observed data and averaging genes imputed multiple times makes the pairwise strategy invariant to the order in which pairs of studies are constructed.

### 2.2 Imputation under incomplete validation set and sparse signals

The pairwise approach must be refined before it can be used for practical applications. The main limitation of the approach as stated is 2-fold: (i) it assumes that all studies are for training and no validation study is present; (ii) all genes that appear in at least one study should be included in the final prediction model. The first assumption makes cross-study validation inaccessible while the second can lead to overfitting and increase the computational cost of the approach if *p_s_* is large. Therefore, we propose the following ‘Core’ and ‘All’ variations of the generic pairwise approach, with ‘Core’ using selected cross-study genes to build the imputation model and ‘All’ using all available genes in the studies. The algorithm statements of the ‘Core’ and ‘All’ variations are provided in Supplementary material S2.3.

#### 2.2.1 ‘Core’ pairwise approach

Before introducing the methods, we make the assumption that the response variable is not available in the validation set. For each study, suppose not all genes are predictive of the outcome, and due to the mixture of signal and noise, a common pre-processing step to filter to a subset of genes that are most related to the outcome is applied. For example, in each training study, we can select the top *q* genes with the largest magnitudes of coefficient estimates from LASSO, where the response is the outcome and the predictors are the expression values of the genes.

The ‘Core’ pairwise approach takes two training sets denoted by $T_{i}$ and $T_{j}$ as a pair, and imputes the missing genes in $T_{i},\;T_{j}$ and the validation set (***V***). In the preliminary screening stage, suppose in each training set that the top *q* predictive genes are selected for the final prediction model. Due to study heterogeneity, different sets of genes may be chosen from the two training sets, and we denote them as $Q_{i}$ and $Q_{j}$, respectively. Furthermore, let $Q_{V}$ be all the available genes in ***V***, $H=Q_{i}\cup Q_{j},\;H_{1}=Q_{i}\cap Q_{j}\cap Q_{V}$ and $H_{2}=H\backslash H_{1}$. The idea of ‘Core’ pairwise approach is to impute the genes in $H_{2}$ that are not shared by all of the three studies using genes in $H_{1}$ that are common across all studies. Note that for the ‘Core’ pairwise approach, the genes used for imputation are all predictive of the outcome in at least one of the training sets. To properly perform ‘Core’ imputation, three different scenarios need to be considered: (i) if a gene is found in only one of $Q_{i}$ and $Q_{j}$ and is also missing in $Q_{V}$, an imputation model will be built in the training set that has this gene available, and imputation will be performed for the other training set and ***V***; (ii) if this gene is not missing in $Q_{V}$, then no imputation is needed in ***V*** since we can use the original values of this gene; (iii) if a gene is available in both $Q_{i}$ and $Q_{j}$ but is missing in $Q_{V}$, then we merge $T_{i}$ and $T_{j}$ together to train a single imputation model for this gene and impute in ***V***. If we have *S *>* *2 training sets, we can repeat the above procedure for all possible $\left(\begin{array}{c} S\\ 2 \end{array}\right)$ pairs of training sets combined with the additional validation set ***V***, and if a gene is imputed multiple times, we take the average over the multiple imputed values as the final imputation.

We provide a more in-depth illustrative example as well as an algorithm statement in Supplementary material S2.

#### 2.2.2 ‘All’ pairwise approach

The ‘Core’ pairwise approach introduced above will only use the genes in $H_{1}$, which are the genes that are predictive of the outcome in training sets, to impute the missing genes in $H_{2}$. However, it is possible that genes not selected for predicting the outcome (i.e. genes not in H) are still helpful for imputing the missing gene expression values. Therefore, another imputation strategy is to use the intersection of all available genes from the three studies instead of focusing only on the intersection of the top predictive genes.

Denote $Q_{i}^{c}$ and $Q_{j}^{c}$ as the other existing genes in $T_{i}$ and $T_{j}$ but not in $Q_{i}$ and $Q_{j}$, and let $H^{c}=Q_{i}^{c}\cup Q_{j}^{c},\;H_{\mathrm{int}}=(Q_{i}\cup Q_{i}^{c})\cap (Q_{j}\cup Q_{j}^{c})\cap Q_{V}$. The idea of the ‘All’ pairwise approach is to use genes in $H_{\mathrm{int}}$ to impute the study-specific missing genes in $H_{2}$. Note that the genes in $H_{\mathrm{int}}$ are the intersection of all the available genes in $T_{i},T_{j}$ and ***V***, and thus not necessarily predictive of the outcome. Four scenarios require consideration (i) if a gene is completely missing (e.g. not in $Q_{i}$ nor in $Q_{i}^{c}$) in one of the training sets and $Q_{V}$, an imputation model will be built for this gene in the training set that has this gene and imputation will be performed for the other training set and ***V***; (ii) if this gene is available in $Q_{V}$, then no imputation is needed in ***V*** since we can use the original values of this gene; (iii) if the gene is found in $H^{c}$ (i.e. this gene is not predictive of the outcome in one of the training sets, but still exists) but is completely missing in $Q_{V}$, the training set that has this gene missing in the top *q* predictive gene list can still use its original value, and then we merge the two training sets together to build a single imputation model for this gene and imputation will be performed in ***V***; (iv) if the gene is found in both $H^{c}$ and $Q_{V}$, all studies will use their original values and no imputation is needed. An illustrative example and algorithm statement are provided in the Supplementary material S2.

## 3 Results

### 3.1 Simulation

#### 3.1.1 Comparison between pairwise and merged approaches

We perform a simulation study to compare the performance of our proposed pairwise approach to the merged approach, where we first merge all studies together and use the intersection of variables across all studies to impute study-specific missing variables. We generate four training studies and one external validation study with sample size of 100 for each study, and we evaluate the performance of the imputation methods in terms of the prediction root mean square error (RMSE) in the validation dataset. The overall RMSE is averaged over 300 simulation iterations.

The data for each study is generated from a model following a similar data generation mechanism as in van Vliet *et al.* (2008):

(1) $$Y=\beta_{1}X_{1}+\cdots +\beta_{5}X_{5}+\beta_{1}^{*}X_{1}^{*}+\cdots +\beta_{5}^{*}X_{5}^{*}+\epsilon ,$$

where

$$\left[\begin{array}{c} X\\ X^{*} \end{array}\right]∼\text{MVN}(0.1,\Sigma )$$

with the variance and covariance being 1 and 0.5, respectively.

To create study-specific patterns of missingness across the four training studies, we fix *X*_1_, *X*_2_ to be common to all studies, while varying the number of missing variables among $X_{3},\ldots ,X_{5},X_{1}^{*},\ldots ,X_{5}^{*}$ across the four training sets. The validation set is complete, and no imputation is needed.

To predict the outcome of interest, we compare the omitting method where only the intersected variables common across all studies are used for predicting the outcome, pairwise linear and polynomial imputation, and merged linear and polynomial imputation. Table 1 summarizes the imputation models for the study-specific missing variables and the final prediction models for each method.

**Table 1.** Imputation methods considered for comparison

Methods	Imputation model	Final predicting model
Omitting	—	LASSO
Pairwise linear imputation	LASSO with linear terms of intersected variables	LASSO
Pairwise polynomial imputation	LASSO with polynomial terms of intersected variables	LASSO
Merged linear imputation	LASSO with linear terms of intersected variables	LASSO
Merged polynomial imputation	LASSO with polynomial terms of intersected variables	LASSO

**Fig. 2.**
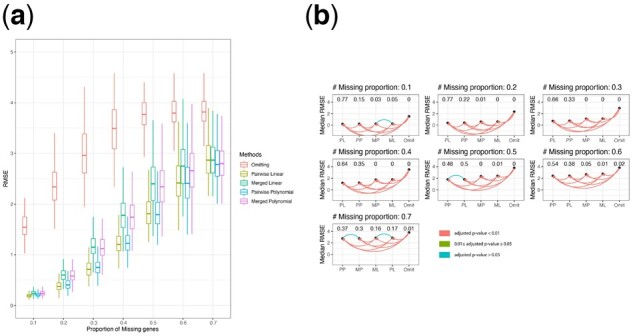
(**a**) The RMSE of prediction on the validation set for different imputation methods and the omitting method. (**b**) Pairwise paired Wilcoxon test of the median RMSE of different imputation methods and omitting method over 300 simulation replicates. ‘PP’, ‘MP’, ‘PL’ and ‘ML’ stand for pairwise polynomial, merged polynomial, pairwise linear and merged linear imputation, respectively. A red line indicates that the Bonferroni adjusted *P*-value from the paired Wilcoxon test is <0.01; a green line indicates that the adjusted *P*-value is >0.01 but <0.05; and a blue line indicates that the adjusted *P*-value is >0.05; and the method to which the arrow is pointing has a significantly smaller median RMSE. The number above each method presents the proportion of times each method has the smallest prediction RMSE in the validation set across the 300 simulation replicates (A color version of this figure appears in the online version of this article)

Figure 2a shows the RMSE of prediction on the validation set from different imputation methods and the omitting method over the 300 simulation iterations. The omitting method consistently has the worst performance of all methods, and the two pairwise imputation methods have relatively better performance than the corresponding merged imputation methods. To formally compare the performance of different methods by accounting for variation across iterations, we performed pairwise Wilcoxon tests on the RMSE. Figure 2b graphically presents the test results, where each method is represented by a single point ordered by the median RMSE over the 300 simulation replicates. The color of the line connecting any two methods indicates the significance level of the test result: a red line indicates that the *P*-value is <0.01; a green line indicates that the *P*-value is >0.01 but <0.05; and a blue line indicates that the *P*-value is >0.05 (*P*-values are Bonferroni adjusted for multiple comparisons). For the cases with *P*-values <0.05, we add a directed arrow to indicate the direction of the test, such that the method to which the arrow points has significantly smaller median prediction RMSE. Above each method, we report the proportion of simulation replicates for which that method obtained the smallest prediction RMSE in the validation set across the 300 simulation replicates. As shown in the figure, when the proportion of missingness is 10–40%, the pairwise linear imputation method has significantly better performance than the other methods, while when the proportion of missingness is 50–70%, the pairwise polynomial imputation method has slightly smaller prediction RMSE. In Supplementary Figure S1a, we plot the log RMSE ratio between different imputation methods and the omitting method: $\text{log}\;\left(\frac{\text{RMSE from the imputation methods}}{\text{RMSE from the omitting method}}\right)$; and Supplementary Figure S1b shows the log RMSE ratio between the pairwise imputation methods and the merged imputation methods. We also show the average difference in the number of intersected variables used to impute the study-specific missing variables between the merged imputation methods and the pairwise imputation methods as the cross-points in Supplementary Figure S1(b). The cross-points show that as the proportion of missing variables increases, the pairwise imputation methods have increasingly larger numbers of intersected genes that can be used to impute the study-specific missing genes as compared to the corresponding merged imputation methods, and the largest discrepancy occurs when the proportion of missing genes is 30%. However, as more genes are missing, the difference approaches 0. This pattern matches with the trend in the log RMSE ratio shown in the same figure, where it initially decreases, but then increases to 0.

Finally, we also consider a scenario that better resembles real gene expression data, where each dataset contains both genes that are predictive of the clinical outcome as well as genes that are irrelevant to the outcome. The data generation mechanism is as follows:

(2) $$\begin{array}{c} Y_{i}=\beta_{1}X_{1,i}+\beta_{2}X_{2,i}+\beta_{3}X_{3,i}+\cdots +\beta_{5}X_{5,i}\\ +\beta_{6}X_{1,i}^{*}+\cdots +\beta_{10}X_{5,i}^{*}\\ +\beta_{11}Z_{1,i}+\cdots +\beta_{20}Z_{10,i}+\epsilon_{i} \end{array},$$

where $X_{1,i},\ldots ,X_{5,i},X_{1,i}^{*},\ldots ,X_{5,i}^{*}$ are generated the same way as in Equation (1), while $Z_{1,i},\ldots ,Z_{10,i}$ follow a multivariate normal distribution with mean 0.1, standard deviation 1 and correlation coefficient 0.2. We restrict the coefficients $\beta_{11}=\beta_{12}=\cdots =\beta_{20}=0$, such that $Z_{1},\ldots ,Z_{10}$ can be regarded as the genes that are irrelevant to the clinical outcome. We also vary the number of missing variables in $Z_{1},\ldots ,Z_{10}$ to explore the performance of the pairwise imputation and merged imputation in the presence of missing irrelevant variables. Detailed simulation results can be found in Supplementary Figure S2, where the pairwise imputation method consistently has a smaller RMSE of prediction on the validation set compared to the merged imputation method regardless of the number of missing relevant or irrelevant genes.

#### 3.1.2 Comparison between the ‘Core’ and ‘All’ pairwise methods

To mimic genomic datasets, we perform another simulation study where the signature genes for predicting the outcome of interest are sparse in the whole dataset. For illustrative purposes, we have two training sets, and we will make predictions on another validation set that also has missing genes. Since Section 3.1.1 suggests that the pairwise strategy generally performs better than the merged strategy in terms of prediction RMSE, in the subsequent simulation study, we provide a focused comparison of the ‘All’ and ‘Core’ pairwise strategies described in Section 2.2.

We generate data as follows, with the sample size of each study set to be 100:

(3) $$\begin{array}{c} Y_{i}=\beta_{1}X_{1,i}+\cdots +\beta_{20}X_{20,i}+\beta_{21}X_{1,i}^{*}+\cdots +\beta_{40}X_{20,i}^{*}\\ +\beta_{41}Z_{1,i}+\cdots +\beta_{160}Z_{120,i}+\epsilon_{i} \end{array},$$

where $X_{1},\ldots ,X_{20},X_{1}^{*},\ldots ,X_{20}^{*}$ jointly follow a multivariate normal distribution with mean 0.1, variance 1 and covariance 0.5. $Z_{41},\ldots ,Z_{120}$ jointly follows a multivariate normal distribution with mean 0, variance 1 and correlation coefficient 0.2. We restrict the corresponding coefficients $\beta_{41},\ldots ,\beta_{160}=0$ such that $Z_{1},\ldots ,Z_{120}$ can be regarded as the genes that are irrelevant to the outcome of interest. In simulation, $X_{1}-X_{20}$ are available to all datasets, and we deliberately set 10 of the genes among $X_{1}^{*},\ldots ,X_{20}^{*}$, and 50 irrelevant genes among $Z_{1},\ldots ,Z_{120}$ to be missing for both the training and validation sets. Therefore, for each study, we have 20 common predictive genes, 10 study-specific predictive genes and 70 study-specific irrelevant genes.

**Fig. 3.**
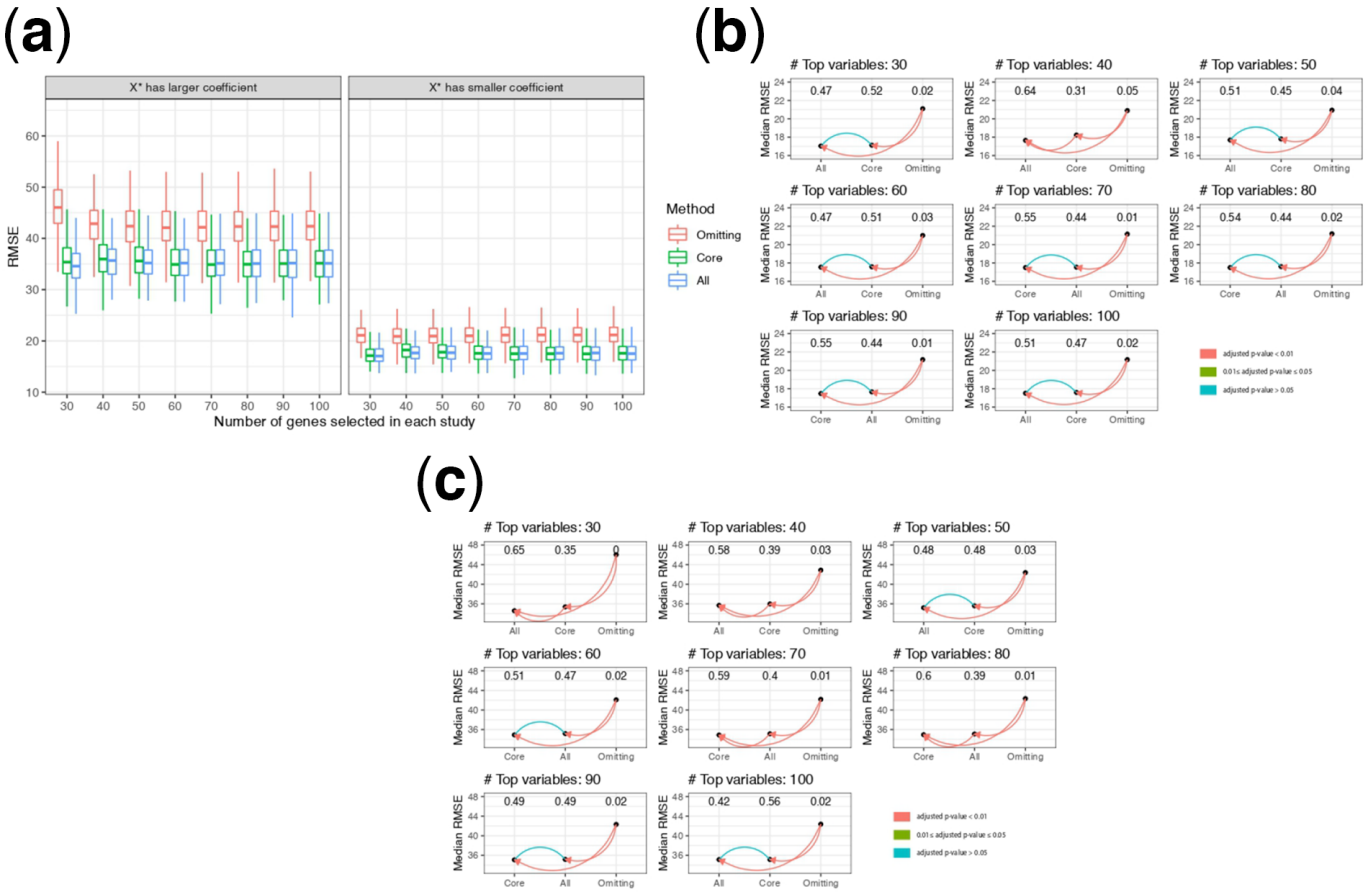
(**a**) RMSE of prediction on the validation set for the Omitting, ‘Core’ and ‘All’ imputation method across the 300 simulation replicates. Left panel: $\beta_{1}=\cdots ,=\beta_{20}=5,\beta_{1}^{*}=\cdots ,=\beta_{10}^{*}=10$; Right panel: $\beta_{1}=\cdots ,=\beta_{20}=10,\beta_{1}^{*}=\cdots ,=\beta_{20}^{*}=5$; (**b, c**) Pairwise paired Wilcoxon test on RMSE between Omitting, ‘Core’ and ‘All’ imputation methods for scenarios when $X^{*}$’s have larger and smaller coefficients than *X*’s, respectively. The *P*-values are adjusted using Bonferroni correction for multiple comparisons

For preliminary feature screening, we applied LASSO and selected the top n,n=30,40,…,100 genes with the largest absolute coeffecients associated with the outcome. Figure 3a shows the RMSE of prediction on the validation set for the omitting, ‘Core’ and ‘All’ imputation methods. Figure 3b and c shows the paired Wilcoxon test results on the RMSE. Supplementary Figure S3a shows boxplots of the log RMSE ratio of the ‘Core’ and ‘All’ imputation methods to the omitting method, and Supplementary Figure S3b shows boxplots of the log RMSE ratio of the ‘Core’ imputation method to the ‘All’ imputation method. Across these figures, both ‘Core’ and ‘All’ imputation methods have better prediction performance than the omitting method. When the number of top genes included is small, ‘All’ imputation has a smaller prediction RMSE than ‘Core’ imputation method, while when the number of genes included is large (>60 or 70), ‘Core’ imputation works better. We hypothesize that LASSO may inevitably include noise among the top predictive genes while some signal will be neglected, and thus when the number of top genes included for prediction is small, ‘All’ imputation has the advantage of access to more informative genes to impute missing genes. However, when the number of genes included is large, most signals will be selected by LASSO and therefore both ‘Core’ and ‘All’ imputation will use approximately the same number of truly informative genes for imputation, while for ‘All’ imputation, more noise will be included in the imputation model, yielding less accurate imputation. In addition, we consider a scenario where $X_{1},\ldots ,X_{20}$ are generated from a multivariate normal distribution but $X_{1}^{*},\ldots ,X_{20}^{*}$ are generated as a complex, non-linear function of $X_{1},\ldots ,X_{20}$, in particular using sine and cosine functions. The simulation results are presented in Supplementary Figures S4 and S5, and we observe similar patterns.

### 3.2 Sensitivity analyses

Apart from using linear regression and polynomial regression as the imputation models, we also explore using more complex machine learning algorithms such as Random Forest, Support Vector Machines and Multiple Imputation. Supplementary Figures S9 and S10 show the prediction RMSE. Regardless of which imputation algorithm is used, the pairwise strategy consistently has smaller prediction RMSE than the corresponding merged approach.

Supplementary Figures S7 and S8 show the prediction RMSE when three studies are used for imputation at a time rather than two. The pairwise strategy consistently has a lower prediction RMSE than imputation across three studies at a time. The difference in performance is due to the pairwise strategy retaining a larger set of intersected features to be used for imputation. As the number of studies used in the subset increases, the subset imputation approach will converge to the merging approach.

We varied the number of training sets used across 3, 6 or 9 training datasets; results are shown in Supplementary Figures S11 and S12. The pairwise strategy consistently has smaller prediction RMSE than the merged approach regardless of the number of training sets. As the number of training sets increases, the intersection used by the merged strategy will shrink while the intersection used for any pair in the pairwise strategy will remain roughly the same size.

**Fig. 4.**
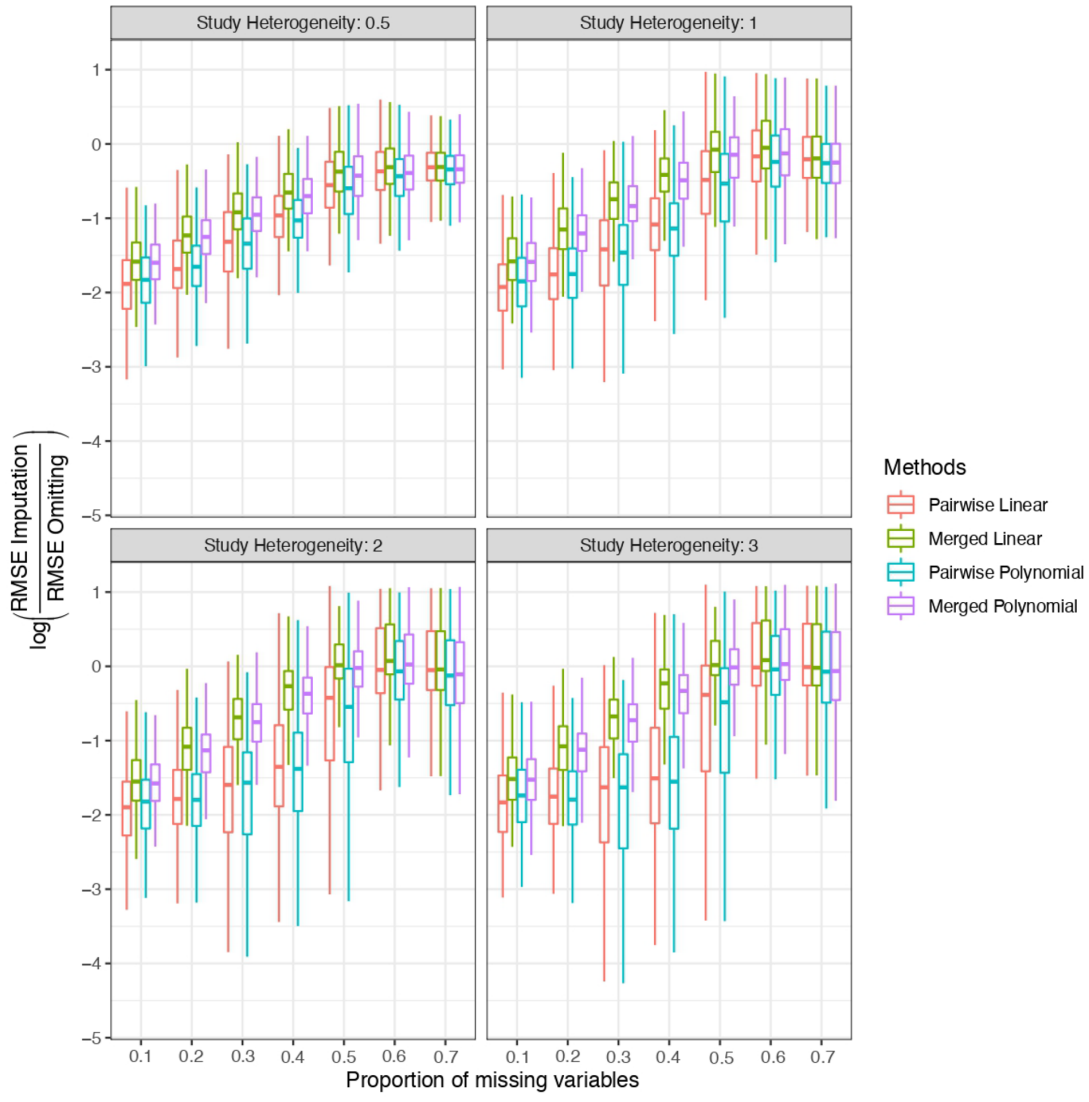
Add study heterogeneity in the $X-X^{*}$ relationship. The baseline method for comparison is omitting

We also assess the impact of cross-study heterogeneity when generating data. For Equation (1), ***X*** is still generated from a multivariate normal distribution with mean 0.1, variance 1 and covariance 0.5. To generate $X^{*}$, we first generate the study-specific mean slopes $\gamma_{k}∼N(0,\tau^{2})$. In the *k*th study, $X_{j}^{*},j=1,\ldots ,5$ is generated as $X\gamma_{k,j}^{T}$, where $\gamma_{k,j}∼\text{MVN}(\gamma_{k},1)$. Therefore, $\tau^{2}$ controls the study heterogeneity of the relationship between ***X*** and $X^{*}$ across studies, and larger $\tau^{2}$ corresponds to more heterogeneous ***X***− $X^{*}$ relationships across studies. For Equation (2), to generate *Z*, we additionally generate the study-specific mean $\mu_{k}∼N(0,\tau^{2})$ for the *k*th study, and $Z_{1},\ldots ,Z_{10}$ are obtained from a multivariate normal distribution with mean $0.1+\mu_{k}$ with variance 1 and covariance 0.2. Figure 4 and Supplementary Figures S13–S15 show the corresponding results. We observe that even under study heterogeneity, both the pairwise and merged strategies have smaller prediction RMSE than the omitting method, and the pairwise strategy consistently has better prediction performance than the corresponding merged strategy. This is attributable to the added robustness of the ensemble approach implemented in the pairwise strategy, where the final imputation is an average of imputations from multiple prediction models. Ensembling in this manner can smooth over cross-study heterogeneity and exceed the advantage of the larger sample size used by the merged model (Guan *et al.*, 2019).

Lastly, we compare the run time of the pairwise imputation and merged imputation strategies. Supplementary Figure S20 shows the run time for linear pairwise and merged strategies when data are generated following Equation (1) across 3, 6 and 9 training studies. Since imputation models will be built multiple times, the pairwise approach takes longer, and this can be exacerbated by the total number of studies.

### 3.3 Real data analysis

We apply the ‘Core’ and ‘All’ pairwise strategies with polynomial imputation to impute study-specific missing genes on microarray datasets from the ‘curatedBreastData’ Bioconductor package (Planey and Butte, 2013). We selected studies numbered 12093, 16446, 17705, 20181, 20194, 2034, 25055 and 25065 because they all used the Affymetrix Human Genome U133A chip for microarray gene expression measurements. The sample sizes of these studies range from 54 to 286, with a total of 1328 patients across studies.

We apply the ‘Core’ and ‘All’ strategies to predict the expression level of the gene ESR1. In each experiment, we take four studies as training sets and a fifth study is chosen as the validation set. The imputation and the final predictive models are all performed using LASSO.

We restrict our analysis to the top 1000 most variable genes in each study. This induces heterogeneous missing patterns across our candidate studies, as the set of 1000 highest-variance genes varies across studies. The distribution of the variability of the genes for each study is shown in Supplementary Figure S16. In general, the variability is similar across studies. Around 35% of the top 1000 variable genes are common across all eight studies, with each study having around 650 study-specific genes that are missing in at least one of the eight studies. We perform a principal component analysis on the intersected genes and plotted the first two principal components annotated by study shown in Supplementary Figure S17. We observe that even after batch effect correction, there still exists some study heterogeneity since there are two distinct clusters with each cluster consisting of four studies.

**Fig. 5.**
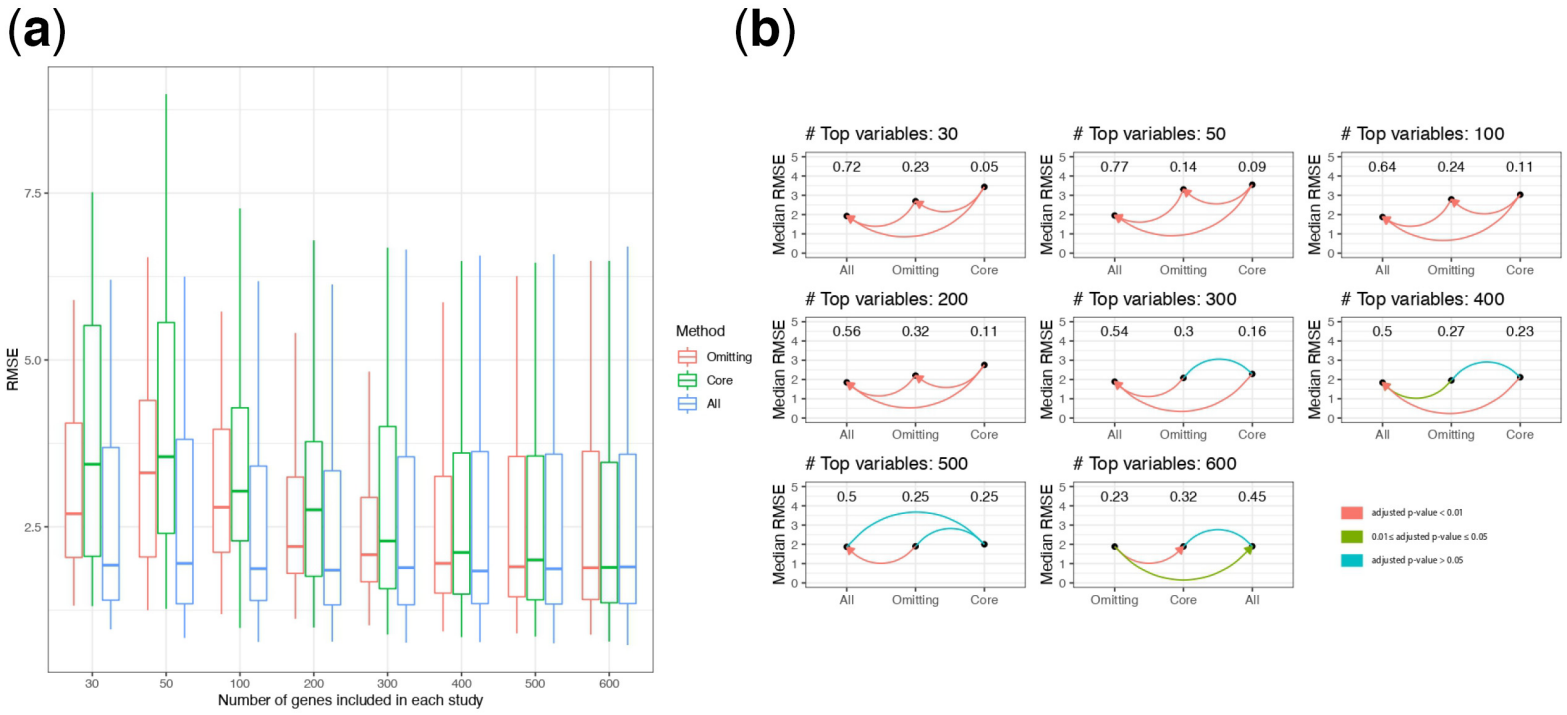
(**a**) RMSE of prediction on the validation set for Omitting, ‘Core’ and ‘All’ imputation methods. (**b**) Pairwise paired Wilcoxon test on the RMSE between Omitting, ‘Core’ and ‘All’ imputation methods

Since not all genes are predictive of the outcome, for the screening step, we fit a LASSO model to predict ESR1 based on other gene expression levels in each study and select the genes with a larger magnitude of coefficients. We then vary the numbers of top predictive genes we select in each study to predict the expression of ESR1. Figure 5a shows the RMSE of prediction on the validation set for the omitting, ‘Core’ and ‘All’ strategies. When the number of top genes selected for predicting the outcome in each study is fewer than 400, the ‘All’ strategy has better performance than the omitting method. But as the number of predictive genes included in each study reaches 600, the RMSE from ‘Core’ and ‘All’ strategies seem to be similar in performance to omitting. Figure 5b shows the paired Wilcoxon test results on the RMSE between the three methods. Contrary to the boxplots of the marginal RMSE in Figure 5a, the ‘All’ strategy consistently has a significantly smaller prediction RMSE than the omitting method. Figure 5b therefore contains the paired information comparing different methods that is not reflected by simply comparing the marginal RMSE of prediction. Supplementary Figure S6a shows the log RMSE ratio of the ‘Core’ and ‘All’ strategies to the omitting method as we vary the number of top predictive genes included for predicting the outcome, and Supplementary Figure S6b shows the log RMSE ratio of the ‘Core’ to ‘All’. Consistent with the paired Wilcoxon test in Figure 5b, we observe that regardless of the number of genes we have included to predict ESR1 expression levels, the median log RMSE ratios of the ‘All’ imputation strategy is always smaller than 0, indicating that more than half of the experiments have a decrease in the RMSE by employing the ‘All’ imputation strategy to account for the study-specific missing genes.

We also vary the number of training sets in each experiment. Supplementary Figures S18 and S19 show the prediction RMSE in the testing set when we use 2 or 7 training sets. We observe that the ‘All’ strategy consistently yields smaller prediction RMSE and is robust against the number of top predictive genes that are included for analysis. The ‘Core’ strategy is less robust and is sometimes worse than the omitting method. This is likely due to ‘All’ using a larger gene set for building imputation models than ‘Core’, using genes that are not predictive of the outcome but still informative for imputing missing genes. When the number of top predictive genes increases to 500 or 600, the three methods have comparable performance. In this scenario, most truly predictive genes are already included in the datasets and imputing the remainder does not impact the eventual prediction model trained.

## 4 Discussion

In this article, we propose a pairwise strategy to apply imputation methods which account for differing feature sets across multiple studies when the goal is to combine information across studies to build a predictive model. Compared with the traditionally convenient method of discarding non-intersected genes or the simpler approach of merging studies together and imputing using genes shared by all studies, our method maximizes common genes for imputation based on the intersection between two studies at a time. Our simulation studies show that the pairwise method has significantly better performance than the omitting and merged methods in terms of the RMSE of prediction on an external validation set. This advantage is more pronounced when there are more studies or when there is cross-study heterogeneity in the inter-gene relationships, and the pairwise method exhibits the best performance no matter the underlying imputation model (e.g. regression, ML and multiple imputation).

Since only a subset of genes are likely to be relevant to the outcome of interest and because the external validation sets may also have genes missing systematically, we also compared ‘Core’ and ‘All’ variations of the pairwise method. Our simulation studies here show that both the ‘Core’ and ‘All’ methods will decrease the RMSE of prediction compared to the omitting method, with ‘All’ demonstrating better performance than ‘Core’, especially when the number of genes included for prediction is small. In our real data examples, ‘All’ imputation again has better performance than ‘Core’ and ‘Core’ imputation tends to be more volatile than ‘All’ imputation. In examining the resulting imputation and prediction models, we find that we are less successful at selecting relevant features using LASSO as we were in simulation when the data-generating mechanism was simple and well defined.

When ‘Core’ imputation is successful, following Spooner *et al.* (2020) we conjecture that this is because using features that are known to be predictive of the outcome across studies to build imputation models is more reliable and robust to cross-study heterogeneity than using a mixture of cross-study and study-specific features (‘All’ imputation). In ‘All’ imputation, it is possible that a study-specific feature which is only coincidentally predictive within that study will replace a more reliable cross-study feature from the intersection, and while the resulting imputation model would exhibit good performance for that study, it may not generalize well to imputing the same missing feature across studies. We echo the conclusion of Spooner *et al.* (2020) that in some cases, feature selection can be more effective than penalization/regularization, and that even if the cross-study selected features are a subset of all features fed to the regularizing model, there may be study-specific features that the regularization prefers.

One limitation of the ‘Core’ and ‘All’ strategies is that neither method is using an optimal gene set to impute the study-specific missing genes. ‘Core’ imputation relies only on genes that are predictive of the outcome while completely neglecting other genes that might be informative of those missing genes even though they are not predictive of the outcome. ‘All’ imputation uses as many genes as possible for imputation with many ‘noise’ genes being included; those additional ‘noise’ features will also lead to less precise imputation of the missing genes. Moreover, an implicit assumption of our imputation procedure is that the genes are missing at random (MAR). If the MAR assumption is violated, for instance, if the missingness mechanism also depends on the outcome, then the imputation might yield even worse predictive performance. We also conduct the bulk of our simulations with a linear model data-generating mechanism, which preserves the interpretability of the induced missingness patterns, but is likely a simplification of practical data-generating mechanisms (however, we do explore more complex associations in the supplement and observe similar patterns).

The observed robustness of the pairwise imputation strategy compared to merging against study heterogeneity in the inter-gene relationships is likely introduced via the averaging approach we implemented to harmonize imputations of the same gene from models trained in different study pairs. This approach can be viewed as a simplified version of the multi-study stacking framework (Patil and Parmigiani, 2018), which utilizes ensemble learning to provide generalizable predictions even in presence of moderate to large study heterogeneity (Guan *et al.*, 2019; Zhang and Ma, 2012). We plan to investigate whether the original multi-study stacking framework can be used to further improve the performance of our imputation strategies as the next steps.

To formally compare the performance of different imputation methods, we applied a pairwise Wilcoxon test on the median RMSE of prediction between different methods. Another future direction of research is on hypothesis tests for rigorous comparison of the performance of different methods that accounts for simulation variation, multiple comparisons and study heterogeneity across multiple studies.

## Supplementary Material

btac839_Supplementary_Data

## Data Availability

The data underlying this article are available in ‘curatedBreastData’ Bioconductor package at https://bioconductor.org/packages/release/data/experiment/html/curatedOvarianData.html.
